# Supplementary material for: Coproduction of marine restoration with communities facilitates stronger outcomes
Source: Camb Prism Coast Futur. 2025 Dec 26;4:e3. doi: 10.1017/cft.2025.10021 (PMC12895437; doi:10.1017/cft.2025.10021)
Supplement: Unsworth et al. supplementary material [file S2754720525100218sup001.docx]

# APPENDIX 1: Time table of key stakeholder engagement events (Taken from Burton et al 2021).

*17^th^ April 2019* – Engagement at Dale Yacht Club, focusing on Crown Estate licence.

*8^th^ July 2019* – Project presentation to Dale Community Council

*23^rd^ July 2019* - Presentation at the Pembrokeshire SAC Joint Relevant Authorities / Welsh Government Marine & Fisheries Division meeting

*26^th^ July 2019* – information stand at Dale Village Fete

*31^st^ July 2019* – Site visit via boat to site for interested local stakeholders followed by project presentation in village hall

*19^th^ August 2019* – Community drop in Information session

*24^th^ August 2019* – Information stand at Village festival

*6^th^ July 2019* – Dale Fort Schools education session

*2^nd^ September 2019* – Shore visit at low spring tide, followed by meeting to discuss alternative locations, and project presentation with extensive Q&A

*24^th^ September 2019* – Community drop-in Information session and local fisher meeting

*7^th^ October 2019* - Community meeting with ‘back to basics’ talk (videoed and made available on YouTube).

*22^nd^ October 2019* – Options workshop for project next steps and questionnaire development in community meeting to help gather wider community perceptions of project and input, followed by questionnaire hand delivery in village the next week. Addendum suggestion made to the Management Scheme

*2^nd^ December 2019* – Community meeting with Dr Geoff Proffitt from Swansea University, who handed out a letter stating the position of Swansea University, including an insurance statement and a draft possible Memorandum of Understanding (MoU). Questionnaire results were presented.

*January 2020* – Schools workshops, filling bags of sand for seeds. PCF’s Community and Climate Change Engagement Coordinator provided an additional activity around greenhouse gas and climate change for three schools.

*24^th^ January 2020* - Pembrokeshire Marine SAC Relevant Authorities Group formally amend the Management Scheme with an addendum specific to the Dale seagrass restoration.

*3^rd^ February 2020* Community meeting agreeing to planting subject to Marine Licence, in the new agreed location.

*12^th^ February 2020* - The Marine License from Natural Resources Wales was granted.

February/March 2020 – Planting. Community members were invited to take part in planting activity, with positive feedback and participation.

*28^th^ April 2020* – online community meeting (via video-conference due to Covid-19 restrictions) to update on planting, to discuss TOR and membership of the Seagrass Stakeholder Group. A pre-meeting was held with members of Dale Yacht Club and the Moorings Officer to discuss the Visitor Mooring agenda point. Project updates continue to be added to the local Peninsular Papers, available on the Marloes village website. These provide a reminder of where to find project information and refer to any meetings held during the period.

*June and July 2020* – Survey and monitoring of the restoration zone and the seeds planted in Feb/March. Ropes cut as agreed in marine license. With Covid-19 restrictions a little bit eased, from July Swansea University were able to complete diving surveys on the 750,000 seeds planted to date.

*14^th^ July 2020* - Dale Seagrass Stakeholder Meeting, chaired in interim by Chair of PCF Board. Agreements included the sign-off of the ToR for the group and the MOU between Swansea University and the Group. It was agreed to deploy 3 visitor mooring buoys on the eastern side of the seagrass meadow zone and 2 small signage marker buoys on the western edge closest to the shore. Funding to maintain these was secured for at least the next 7 years. Buoys were to be deployed to help people avoid any disturbance of the seagrass. Signage buoys were modelled on the successful [Caldey Marine Code buoys](https://www.pembrokeshiremarinecode.org.uk/marine-code-buoys-at-caldey-island/), as deployed by PCF.

*October 2020* - Swansea University, with some local volunteer divers, were able to complete further planting in the restoration area completing well over 1 million seeds planted in Dale Bay.

*23rd November 2020* - Dale Seagrass Stakeholder Meeting. Local Chair voted in to take over at the next meeting. Secretary to be confirmed.

December 2020

The PCF local seagrass [webpage](https://www.pembrokeshirecoastalforum.org.uk/seagrassoceanrescue/) has been simplified, and an agreement made with Dale Seagrass Stakeholder Group to continue to host the latest update, meeting notes and slides as well as contact information.

The visitor mooring and seagrass zone positions have been incorporated into the MHPA leisure guide, including the map in their tide tables. This will be a major conduit for project information to reach wider users of the waterway. Further promotion can happen through i.e. the Dale Yacht Club website. An information sheet on seagrass within the waterway, including the Dale seagrass restoration, has been sent to all mooring holders within the Milford Haven waterway.

# APPENDIX 2: Information Dissemination Summary (Taken from Burton et al. 2021)

## Webpage

A project specific webpage was created for the general public to learn more about the project, provide feedback and as a collation point for all concerns raised. The project information website is hosted by Pembrokeshire Coastal Forum: <https://www.pembrokeshirecoastalforum.org.uk/seagrassoceanrescue/> and outlined the scope of the project and its benefits.


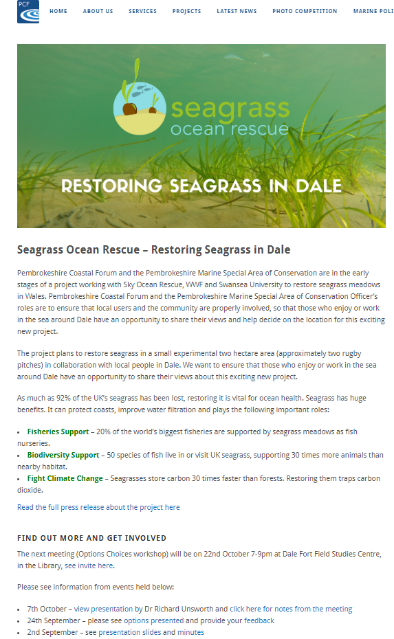
All communicated concerns were collated (updated after each event) to identify questions and provide answers, reassurances and justification in the FAQs section (12 pages).

The website included a feedback form seeking views on the nature of the project, its location, ongoing engagement and information requirements.

Analytics (dated 4/10/19) showed 374 unique pageviews to the Seagrass Ocean Rescue information webpage on the PCF website.

74% straight to the seagrass page (likely via link shared).

The PCF local seagrass [webpage](https://www.pembrokeshirecoastalforum.org.uk/seagrassoceanrescue/) was simplified following the Dale Seagrass Stakeholder meeting in November 2020, and an agreement was made for PCF to continue to host the latest project update, meeting notes and slides as well as contact info for Dr Richard Unsworth, Swansea University and Dale Seagrass Stakeholder Group Chair Fil Marshall.

## Display boards


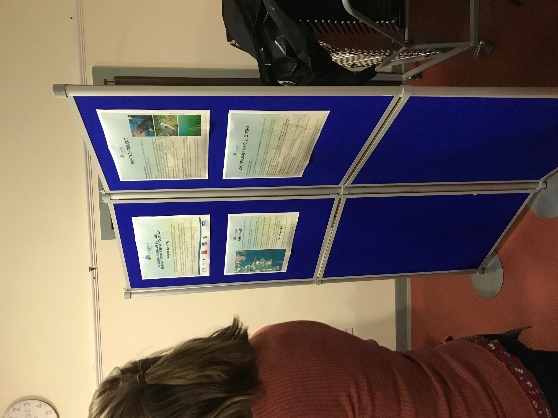
Provided for public events (drop-ins, meetings) with the ability to be flexible according to the focus necessary.


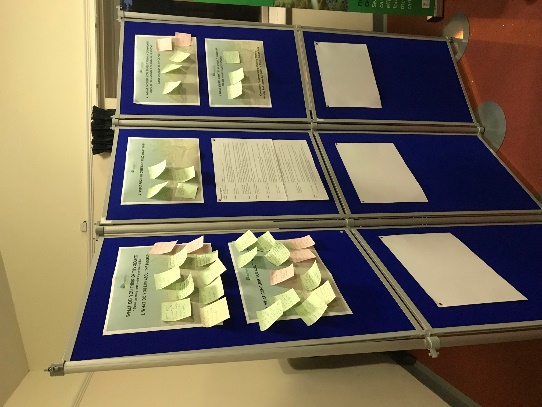


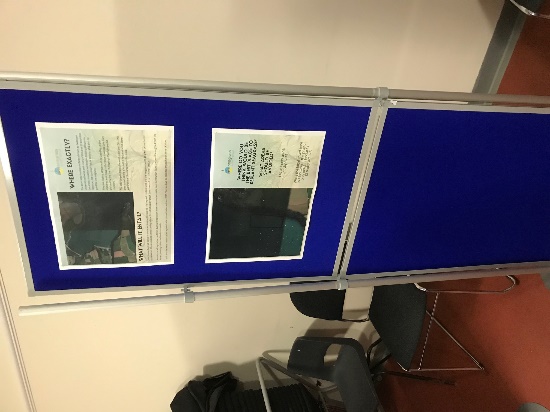


##
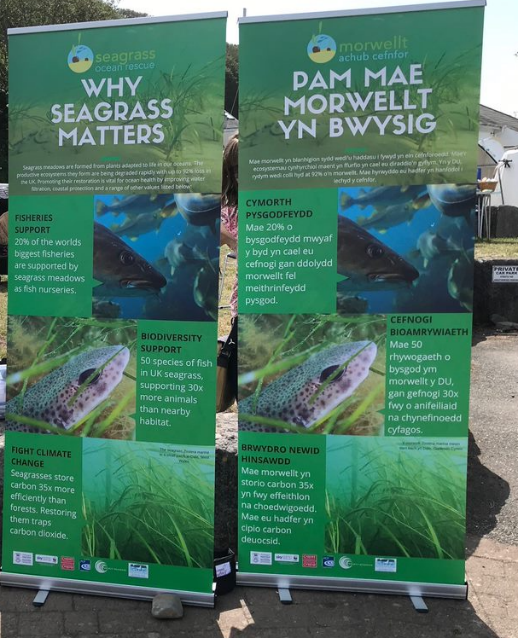
Pop-ups

Providing eye-catching interpretive material is useful for events such as fetes in order to attract interest and entice a conversation where more detail can be provided.


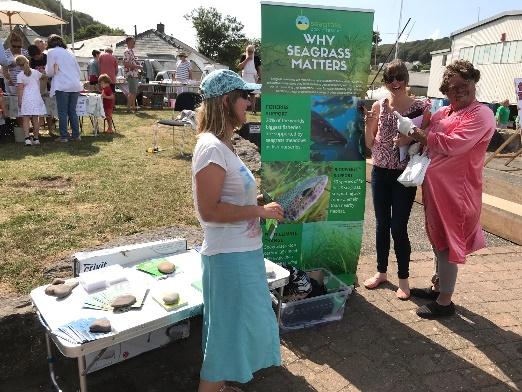


***26/7/2019 Fete***

### Direct engagement with local stakeholders

- Invites via email (and post when someone expressed not being on email) to mailing list (90 people) for all public events listed, these included a link to information page on PCF website.
- Hardcopy letters were sent out in order to contact particular stakeholders not engaged through online or email forums.
- Poster invites to all meetings/events on village noticeboard next to the community hall, in the Dale Yacht Club building, and on the pub wall.
- Regular project updates in *Peninsular Papers* - the local monthly community newsletter available on the Marloes village website and posted in hard copy to every household and business in Dale, Marloes and St Brides, St Ishmaels and Herbranston. Hard copies were not printed and hand delivered for much of the year in 2020 due to Covid-19 restrictions but resumed in August. The double month edition for August/September 2020 included a page and a half seagrass update with photos. Updates provide a reminder of where to find project information and refer to any meetings held during the period. The update for the November/December2020 edition included a map with information about the visitor moorings.
- Direct communication with Dale Community Council.
- Direct communication with Dale Yacht Club.
- Direct communication with principle fisher.
- Phone calls followed by one to one meeting with main mooring servicing organisation and local business
- Phone calls with community councillor and commercial boat operators to update and engage directly
- Email and phone conversations addressing concerns or offering support with stakeholders as required.
- Community Feedback Questionnaire
- Events and site visits.


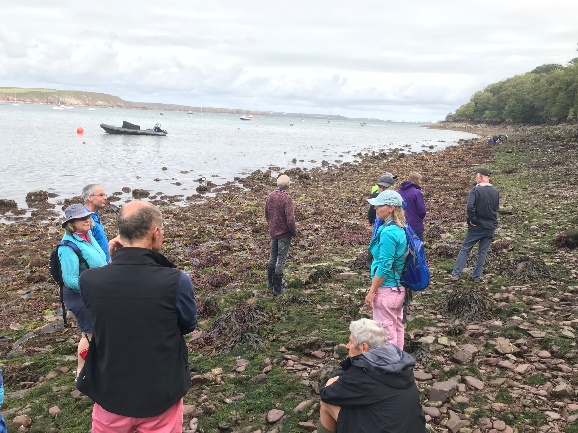


***2/9/2019 Shore Site Visit***


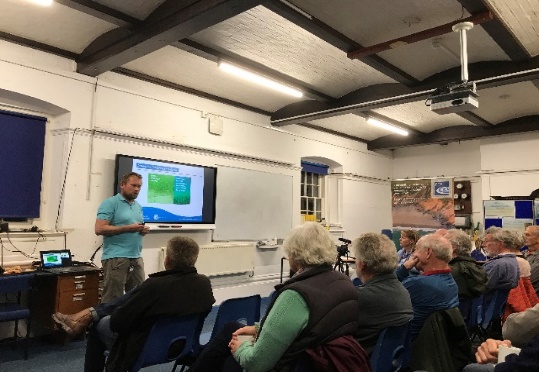


***11/10/2019 Presentation, Q&A***
